# Updated recommendations on the therapeutic role of extracorporeal shock wave therapy for peyronie’s disease: systematic review and meta-analysis

**DOI:** 10.1186/s12894-023-01320-8

**Published:** 2023-09-12

**Authors:** Xiaofeng Wang, Hongquan Liu, Gonglin Tang, Gang Wu, Yongli Chu, Jitao Wu, Yuanshan Cui

**Affiliations:** Department of Urology, Qindao University Medical College Affiliated Yantai Yuhuangding Hospital, No. 20 East Yuhuangding Road, Yantai, Shandong 264000 China

**Keywords:** Extracorporeal shock wave therapy, Meta-analysis, Peyronie’s Disease, Randomized controlled trial

## Abstract

**Background:**

The therapeutic role of extracorporeal shockwave therapy (ESWT) for Peyronie’s disease (PD) has been controversial in a long term. We aimed to further evaluate the therapeutic effect of ESWT for PD on the basis of available high-quality studies.

**Methods:**

The PubMed, CENTRAL and Embase databases were searched for articles published from January 1st, 2000 to December 31, 2022. Only randomized controlled trials (RCTs) using ESWT to treat PD were included. Meta-analysis and forest plots were carried out using Review Manager 5.4.1 software, and outcomes were reviewed by 2 authors independently. Using the Risk of Bias assessment form (ROB-2) by Cochrane Collaboration for quality assessment. PRISMA 2020 guidelines were used in this article to achieve the quantitative and qualitative synthesis of data.

**Results:**

A total of four RCTs were included. 151 patients in the ESWT group and 150 patients in the control group. The meta-analysis results showed that ESWT could significantly reduce plaque size (OR 2.59, 95%CI 1.15 to 5.85, P = 0.02) and relieve pain (MD -1.55, 95%CI -2.46 to -0.64, P = 0.0008); but it has no significant effect on reducing the penile curvature (OR 1.93, 95%CI 0.87–4.26, P = 0.11) and improving sexual function (MD 2.6, 95%CI -1.63 to 6.83, P = 0.23), there is also no significant difference in complication rates between groups (OR 2.94, 95%CI 0.66 to 13.03, P = 0.16). The risk of bias of results is low. The limitations of this study are that the number of included studies is too small, some experimental outcomes are missing, and the expression of outcomes is not unified.

**Conclusions:**

For PD, ESWT can be considered as a safe short-term treatment, which can reduce plaque size and relieve pain, but cannot improve penile curvature and sexual function. Its long-term efficacy remains to be discussed.

**Registration number:**

PROSPERO (ID: CRD42023436744).

**Supplementary Information:**

The online version contains supplementary material available at 10.1186/s12894-023-01320-8.

## Background

Peyronie’s disease (PD) is an abnormal fibrotic disease of the tunica albuginea that manifests as the local formation of fibrotic plaques in the penis. [1] The disease causes penile deformities such as curve and shortening, accompanied by sexual intercourse disorder and pain, [2] its prevalence rate is about 3–9% worldwide, and its impact is more significant for men over 40 years old. [2–4] The disease also affects the quality of life (QoL) of patients and their partners, causing depression. [5] The pathophysiological mechanism of its pathogenesis is still unclear. One widely accepted theory is that repeated sexual intercourse causes slight damage to the tunica albuginea, leading to fibrin deposition, inducing chronic inflammation, and ultimately leading to penile deformity. [1, 6] The uncertainty of the pathogenesis leads to the diversification of treatment options.

Surgical intervention is the main method to correct penis deformity and improve symptoms, [5] but it carries the risks associated with postoperative penile shortening as well as intraoperative anesthesia. [7] Extracorporeal shockwave therapy (ESWT) is a safe nonsurgical modality of treatment that has been used for the first time since 1989. [8] There is evidence that ESWT promotes wound healing in soft tissues. [9] There are multiple hypotheses about the mechanism of its therapeutic effect, the first hypothesis indicates that ESWT may act by directly disrupting and remodeling plaques; the second hypothesis proposes that ESWT may generate heat locally, leading to an active inflammatory response with increased macrophage activity and subsequent plaque dissolution and absorption. [10].

Previous studies have shown that the use of ESWT provides short-term relief of symptoms such as pain and sexual intercourse disorder, but controversy exists regarding the long-term improvement effect of penile curvature as well as plaque. [10–15] And assessment of the long-term efficacy of ESWT and the progression of the disease course in the stable stage of PD is currently lacking. [16–18] Only two meta-analyses of this study have been published in recent years, but their evaluation criteria and final conclusions were not consistent, and the included articles were of low quality with insufficient level of evidence and did not clearly state the applicability of ESWT in PD patients. [19, 20] The therapeutic effect of ESWT on some symptoms remains to be verified.

Our aim is to use the updated clinical trial data to reevaluate the efficacy of ESWT in reducing penile curvature, reducing plaque size, improving sexual function and alleviating pain in PD patients compared with placebo (sham treatment), and to assess the risk of complications.

## Methods

We predefined the objectives and methods of this systematic review and meta-analysis in a protocol registered at PROSPERO (ID: CRD42023436744) and followed the PRISMA 2020 statement.

### Inclusion criteria

Inclusion criteria was RCTs with full text, studies only investigating the effect of ESWT alone compared with placebo or sham treatment on PD patients. Non-English articles and duplicate articles were excluded, other excluded articles were: meta-analysis, systematic review, studies not related to PD, studies including other combined therapies, single-arm studies, conference records, animal experiments, editing replies, uncompleted clinical studies.

### Source of study

The PubMed, CENTRAL and Embase databases were searched for articles published from January 1st, 2000 to December 31, 2022. We performed a systematic search using keywords: “Peyronie’s disease”, “Peyronie disease”, “ESWT”, “Extracorporeal shock wave therapy”, “shock wave therapy”. The search formula was “(Peyronie’s disease OR Peyronie disease) AND (Extracorporeal shock wave therapy OR ESWT OR shock wave therapy)”. Two authors reviewed independently; disagreements were resolved by discussion.

### Data extraction

Data were extracted by two authors. Basic information: author, publication date, journal name, article title. RCTs information: inclusion and exclusion criteria, demographic data of the participants, number of participants in each group, duration of follow-up. Methodological information: intervention of experimental group, intervention control group, artificial erection measures, penile curvature measurement, determination of plaque size and site, penile length measurement, sexual function evaluation, pain assessment, quality of life assessment. Outcomes information: changes in penile curvature, changes in plaque size, changes in pain scores, changes in sexual function scores, and complication rates.

### Data synthesis and analysis

Two authors independently used the ROB2 tool of the Cochrane Collaboration Network to assess the risk of bias. Review Manager 5.4.1 software (The Cochrane Collaboration, Copenhagen, Denmark) was used for statistical analysis. The primary outcomes were the proportion of patients with penile curvature reduction and the proportion of patients with plate reduction. The secondary outcomes were the proportion of patients with pain relief, the proportion of patients with improved sexual function, and the complication rate. Continuous data and dichotomous data were analyzed. Using fixed effect model, results of dichotomous variables were described by odds ratio (OR) and results of continuous variables were described by mean difference (MD). Under the guidance of Cochran handbook, the Mantel–Haenszel Method was used to assess Odds Ratio of dichotomous variables, and inverse-variance method was used to assess mean difference of continuous variables. All results are presented with 95% confidence intervals (CIs). Significance was set at P value < 0.05. The continuous quantitative data was summarized and presented in tables, and missing data was transformed using statistical formulas for supplement. Heterogeneity was tested by I^2^ – test, if I^2^ > 50%, the random effect model was used. Sensitivity analysis of meta-analysis results was performed using the elimination method.

## Results

### Literature search

**Fig. 1:**
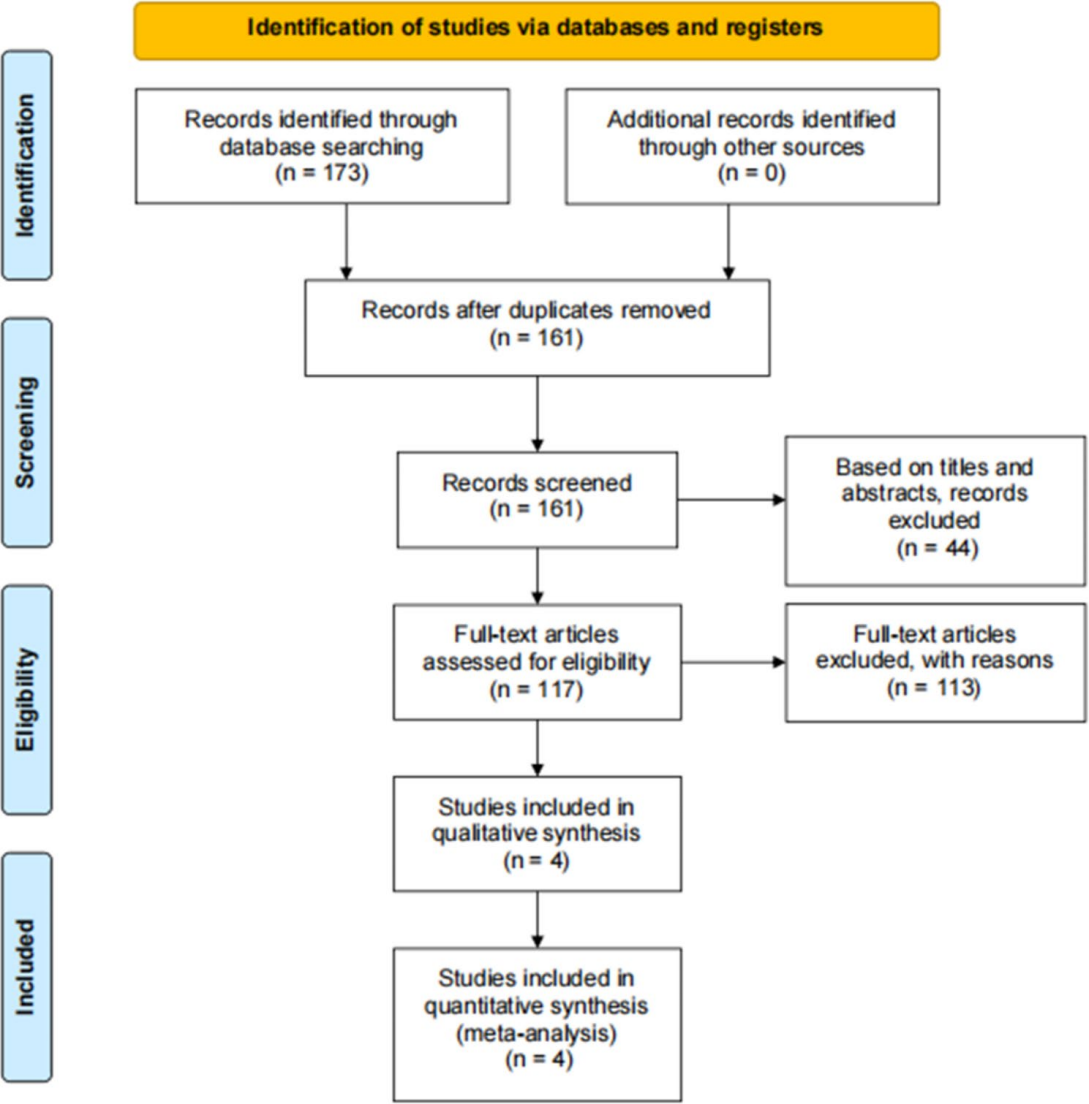
PRISMA data flow diagram for systemic search of databases

173 articles were obtained by searching PubMed, CENTRAL and Embase databases, from which 4 RCTs with high quality were selected for full-text review. [21–24] Other studies that did not meet the requirements of evidence-based medicine were excluded, and the flow diagram is shown in Fig. 1.

### Study characteristics

**Fig. 2:**
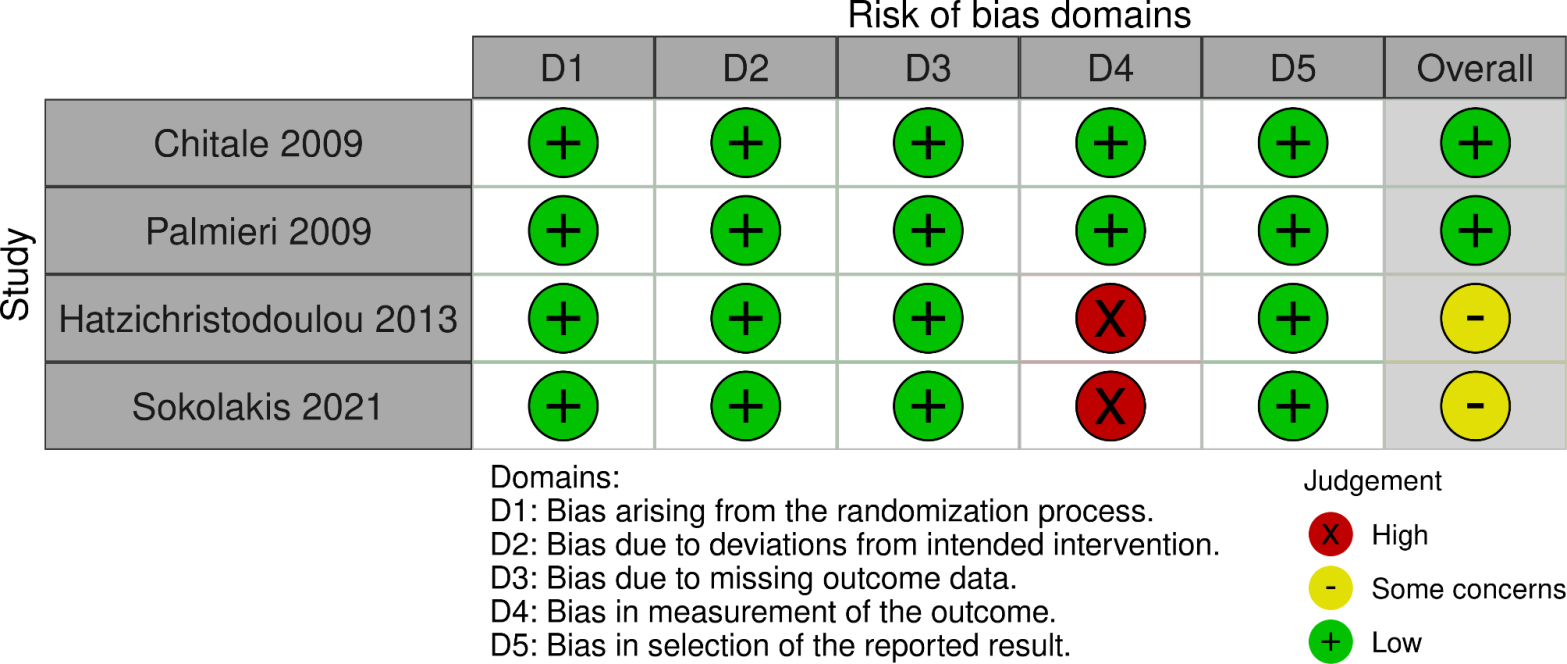
Risk of bias of the included studies

**Fig. 3:**
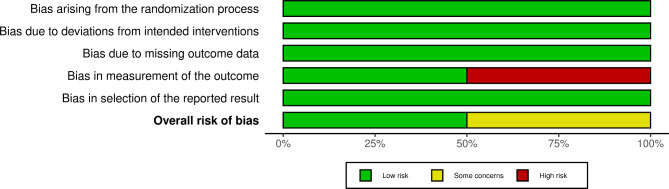
Summary of risk of bias

The baseline characteristics and some of the results of the included studies are shown in Tables 1, 2 and 3. We summarized the methodological characteristics, demographic characteristics, and the continuous quantitative data of the included studies. Using the Risk of Bias assessment form (ROB-2) by Cochrane Collaboration for assessment, the four included studies had a low risk of bias. The results of the risk of bias assessment of individual studies as well as summary are shown in Figs. 2 and 3. Only four articles were included, and a funnel plot was not performed to assess the risk of publication bias because the published relevant studies did not reach a consistent and definite conclusion on the efficacy of ESWT for PD. None of the included studies had a conflict of interest.

**Table 1:** Summary of the methodologies of the included studies

	Palmieri 2009	Chitale 2009	Hatzichristodoulou 2013	Sokolakis 2021
**Design**	RCT	RCT	RCT	RCT
**Blinding**	Double	Double	Single	Single
**Inclusion criteria**	Disease not > 12 months, patient age between 18–75 years, only one plaque demonstrated by basal and dynamic sonography and by palpation with a maximum size of 3.75 cm2, no previous medical or surgical therapies for PD, stable sexual relationship, presence of painful erections (score ≥ 5 on a visual analog scale [VAS] with a score ranging from 0–10), ED, and penis recurvatum.	Stable penile deformity secondary to PD affecting their ability to perform sexual intercourse and/or quality of life due to penile angulation; recent onset of painless deformity of the penis on erection, and stable for > 6 months; pain and/or angulation of the penis on erection; difficult intercourse due to penile curvature, and partner dissatisfaction; a degree of ED (partial) associated with penile deformity; palpable plaque along the penis with penile deformity; aged > 18 years.	Previous unsuccessful oral medical therapy, patient age ≥ 18 years, and plaques and/or pain at erection and/or deviation; disease duration ≥ 12 months and additionally unchanged symptoms (deviation, pain, and plaques) for ≥ 3 months. Oral medical therapy was defined unsuccessful when there was no improvement in pain or deviation.	Male patients ≥ 18 years old; PD lasting for ≥ 12 months; thepresence of penile plaque or pain at erection or curvature; previous unsuccessful oral PD therapy; stable symptoms for ≥ 3 months.
**Exclusion criteria**	Take drugs for ED or other therapies for PD during the course of the study; take analgesics before, during, or after painful erections; patients with blood coagulation disorders, cardiac pacemaker, lower urinary tract infections, and vascular disorders in the path of the shock waves.	Congenital curvature of the penis; previous treatment for PD (surgical/medical); patient on warfarin; patient with total ED in need of therapy for ED.	Prior penile surgery and ED not responding to phoshodiesterase-type-5 inhibitors or intracavernous injections.	Prior penile surgery or ESWT; ED not responding to phosphodiesterase-type five inhibitors or intracavernosal injections; unwillingness or inability to provide informed consent.
**Artificial erection measures**	Intracavernous injection of alprostadil	Intracavernous injection with prostaglandin E1	Alprostadil	NM
**Measure of angle**	Goniometer from three angles (frontal, lateral, and above) by photographic pictures	Angle measure /ruler	Goniometer after artificial erection	Goniometer after artificial erection
**Measure of length**	NM	Flexible pipe cleaner	NM	NM
**Measure of plaque site**	Fully stretched penis during flaccidity by palpation	NM	Palpation and sonography using a 7.5-MHz linear transducer.	Palpation and ultrasound
**Measure of plaque size**	The product of length and width in cm2	NM	Ruler, in mm2	Palpation and ultrasound
**Measure of sexual function**	IIEF-5 score	IIEF-5 score	Self-made scale	The ability to perform sexual intercourse
**Measure of pain**	VAS score	VAS score	VAS score	VAS score
**Quality of life**	QOL score	GAQ score	NM	NM
**Complications**	Bruising	Bruising	Bruising	NM
**Interventions of ESWT group**	Storz Duolith ESWT system	NM	Piezoson 100 lithotripter	Piezoson 100 lithotripter
**Shock waves/energy flux density (mJ/mm2)/emission frequency (Hz)**	2000 shockwaves/0.25 mJ/mm2/4 Hz	3000 shockwaves/level 25 (38 MPa)	2000 shock waves/0.29 mJ/mm2/3 Hz	2000 shockwaves/0.29 mJ/mm2/3 Hz
**Interventions of control group**	Placebo therapy(nonfunctioning transducer)	Sham therapy (3000 SWs /level 0)	Placebo(interposition of a plastic membrane in the transducer)	Sham therapy (interposition of a plastic membrane in the transducer)
**Number of ESWT group**	50	16	51	34
**Number of control group**	50	20	51	29
**Drop out**	0	3	0	39
**Number of sessions**	4	6	6	6
**Frequency of treatment (/week)**	1	1	1	1
**Duration of treatment (weeks)**	6	4	6	6
**Follow-up**	12/24 weeks	6 months	Median of 4 weeks (range 4–26 weeks)	4 weeks/3 years

RCT: randomized controlled trial; NM: not mentioned

**Table 2:** Demographic data of included patients

	Palmieri 2009	Chitale 2009	Hatzichristodoulou 2013	Sokolakis 2021
**Age (mean years (SD))**	S 54 (13)	S 57.8 (8.0)	S 53.8 (11.75)	S 56.4 (10.2)
	C 55.2 (10)	C 60.0 (10.5)	C 55.2 (10.5)	C 57.2 (7)
	P NM	P NM	P > 0.05	P > 0.05
**Duration of symptoms (mean months (SD))**	S 8.74 (1.75)	S 14.9 (8.4)	S NM	S 24 (NM)
	C 8.62 (1.75)	C 32.3 (28.0)	C NM	C 24 (NM)
	P NM	P NM	P NM	P > 0.05

S: study; C: control; P: P-Value; NM: not mentioned

**Table 3:** Summary of continuous quantitative outcomes of the included studies

Outcome	Palmieri 2009	Chitale 2009				Hatzichristodoulou 2013	Sokolakis 2021				
**Pretreatment**											
Penile curvature (Mean (SD))	S 28.9 (6.68)	Dorsal		S 24.9 (11.9)		S 44(NM)	Dorsal			S NM	
	C 29.45 (7.26)			C 33.3 (15.9)		C 43(NM)				C NM	
	P > 0.05			P > 0.05		P NM				P NM	
		Lateral		S 20 (15.3)			Lateral			S NM	
				C 23.7 (20.6)						C NM	
				P > 0.05						P NM	
Plaque size (Mean (SD))	S 1.5 (0.74)	S NM				S NM	S NM				
	C 1.59 (0.71)	C NM				C NM	C NM				
	P > 0.05	P NM				P NM	P NM				
Sexual function (Mean (SD))	S 14 (5)	S 19.3 (6.1)				S NM	S NM				
	C 14.16 (4.75)	C 15.6 (7.9)				C NM	C NM				
	P > 0.05	P > 0.05				P NM	P NM				
Pain (Mean (SD))	S 5.51 (2)	S 1.5 (2.3)				S 4 (1.5)	S NM				
	C 5.19 (2)	C 1.2 (2.3)				C 4 (1.75)	C NM				
	P > 0.05	P > 0.05				P NM	P NM				
**Post-treatment**											
Penile curvature (Mean (SD))	12w S 27.47 (7.75)	Dorsal	S 25.8 (12.6)			S 35 (NM)	Dorsal		S NM		
	C 30.4 (6.62)		C 28.0 (12.8)			C 38 (NM)			C NM		
	P > 0.05		P > 0.05			P NM			P NM		
	24w S 27.45 (8.11)	Lateral	S 20.9 (16.5)				Lateral		S NM		
	C 31.25 (6.74)		C 20.2 (18.5)						C NM		
	P < 0.05		P > 0.05						P NM		
Plaque size (Mean (SD))	12w S 1.46 (0.75)	S NM				S NM	S NM				
	C 1.66 (0.66)	C NM				C NM	C NM				
	P > 0.05	P NM				P NM	P NM				
	24w S 1.44 (0.76)										
	C 1.73 (0.65)										
	P < 0.05										
Sexual function (Mean (SD))	12w S 19.56 (NM)	S 19.9 (4.8)				S NM	S NM				
	C 14.46 (NM)	C 15.7 (7.5)				C NM	C NM				
	P < 0.001	P > 0.05				P NM	P NM				
	24w S 19.4 (NM)										
	C 14.74 (NM)										
	P < 0.001										
Pain (Mean (SD))	12w S 1.6 (NM)	S 0.5 (0.8)				S 1.5 (1.5)	S NM				
	C 4.97 (NM)	C 0.4 (0.7)				C 3 (1.75)	C NM				
	P < 0.001	P > 0.05				P NM	P NM				
	24w S 0.46 (NM)										
	C 2.66 (NM)										
	P < 0.001										
**Change in same group**											
Penile curvature (Mean (SD))	12w S -1.43 (7.27)	Dorsal			S 0.9(16)	S -9 (NM)	4w Dorsal				S -2.1 (NM)
	C 0.95 (6.96)				C -5.3 (11.6)	C -5 (NM)					C 0.2 (NM)
	P (NM)				P > 0.05	P NM					P NM
	24w S -1.45 (7.5)	Lateral			S 0.9 (17.4)		4w Lateral				S 7 (NM)
	C 1.8 (7.01)				C -3.5 (17.4)						C 2.5 (NM)
	P (NM)				P > 0.05						P NM
							3y Dorsal				S 5.9 (NM)
											C 0.6 (NM)
							P NM				
							3y Lateral S 9.3 (NM)				
							C 2 (NM)				
							P NM				
Plaque size (Mean (SD))	12w S -0.04 (0.75)	S NM				S NM	S NM				
	C 0.07 (0.69)	C NM				C NM	C NM				
	P (NM)	P NM				P NM	P NM				
	24w S -0.06 (0.75)										
	C 0.14 (0.68)										
	P (NM)										
Sexual function (Mean (SD))	12w S 5.56 (NM)	S 0.6 (2.6)				S NM	S NM				
	C 0.3 (NM)	C 0.1 (3.3)				C NM	C NM				
	P (NM)	P > 0.05				P NM	P NM				
	24w S 5.4 (NM)										
	C 0.58 (NM)										
	P (NM)										
Pain (Mean (SD))	12w S -3.91 (NM)	S -1 (1.8)				S -2.5 (1.5)	4w	S 3.2 (NM)			
	C -0.22 (NM)	C -0.8 (1.8)				C -1 (1.75)		C 1.3 (NM)			
	P (NM)	P > 0.05				P NM		P < 0.05			
	24w S -5.05 (NM)						3y	S 3.3 (NM)			
	C -2.53 (NM)							C 1.2 (NM)			
	P (NM)							P < 0.05			

S: study; C: control; P: P-Value; W: weeks; NM: not mentioned

### Outcome measures

#### Reduction of penile curvature

**Fig. 4:**
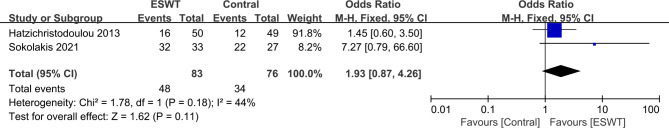
Forest plot of reduction of penile curvature. CI, confidence interval. ESWT, extracorporeal shock wave therapy

The results of treatment were evaluated according to the changes of the penile curvature of the penis in all directions before and after treatment. Two studies with a total of 159 males were included. [23, 24] The results showed that 57.8% (48/83) of the patients in the ESWT group and 44.7% (34/76) of the patients in the control group improved their penile curvature, but there was no statistically significant difference between groups (OR 1.93, 95%CI 0.87 to 4.26, P = 0.11). The heterogeneity of the result was high (I^2^ = 44%, P = 0.18). The meta-analysis result of the proportion of people with improved penile curvature is shown in Fig. 4.

### Reduction of plaque size

**Fig. 5:**
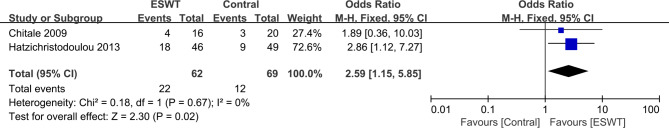
Forest plot of reduction of plaque size. CI, confidence interval. ESWT, extracorporeal shock wave therapy

The treatment effect was evaluated according to the change of plaque size before and after treatment. Two studies with 131 males were included. [22, 23] The results showed that the plaque size of 35.5% (22/62) of patients in the ESWT group and 17.4% (12/69) of patients in the control group decreased, difference between groups was considered statistically significant (OR 2.59, 95%CI 1.15 to 5.85, P = 0.02). There was no heterogeneity in the study result (I^2^ = 0%, P = 0.67). The meta-analysis result of the proportion of people with reduced penis plaque size is shown in Fig. 5.

### Improvement of sexual function

**Fig. 6:**
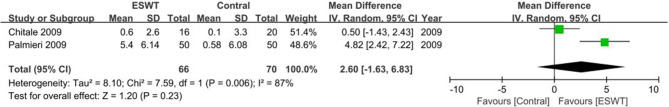
Forest plot of improvement of sexual function. CI, confidence interval. ESWT, extracorporeal shock wave therapy

Sexual function was assessed by **5**-item International Index of Erectile Function (IIEF-5) score or a self-made scoring scale, treatment effects were assessed according to changes of scores before and after treatment (increase of score indicated improvement of sexual function). Two studies involving 136 males with IIEF-5 scores were included. [21, 22] The results showed that there was no significant difference between the two groups (MD 2.6, 95%CI -1.63 to 6.83, P = 0.23). There was high heterogeneity in the study result (I^2^ = 87%, P = 0.006). The meta-analysis result of the improvement of sexual function represented by IIEF-5 score changes is shown in Fig. 6.

### Relief of pain

**Fig. 7:**
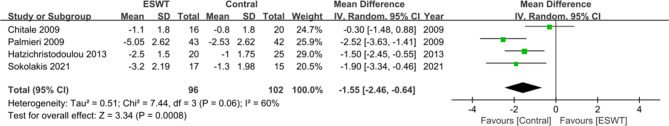
Forest plot of relief of pain. CI, confidence interval. ESWT, extracorporeal shock wave therapy

The level of pain in the patients was assessed by VAS (visual analogue scale) scores, and the effect of treatment was assessed by the change of scores before and after treatment (score reduction indicated pain relief). Four studies with 198 males were included. [21–24] There was a significant difference between the two groups (MD -1.55, 95%CI -2.46 to -0.64, P = 0.0008). There was high heterogeneity in the study result (I^2^ = 60%, P = 0.06). The meta-analysis result of pain relief represented by VAS score changes is shown in Fig. 7.

### Rate of complications

**Fig. 8:**
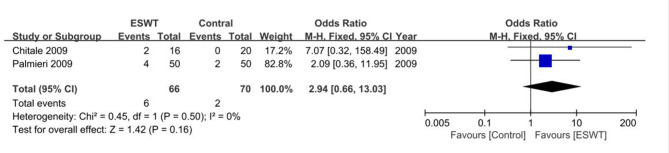
Forest plot of rate of complications. CI, confidence interval. ESWT, extracorporeal shock wave therapy

For homogeneity of the study, we limited the range of complications to bruises on the penile skin surface, excluding smaller bleeding points and friction injuries. Two studies with a total of 136 males were included. [21, 22] The results showed that bruises occurred in 9.1% (6/66) of patients in the ESWT group and 2.9% (2/70) of patients in the control group, with no statistically significant difference between groups (OR 2.94, 95%CI 0.66 to 13.03, P = 0.16). There was no heterogeneity in the result (I^2^ = 0%, P = 0.50). The result of meta-analysis of the proportion of complications is shown in Fig. 8.

### Sensitivity analysis

Sensitivity analysis was performed on the results of meta-analysis of pain relief. The results of the 4 RCTs eliminated one by one showed that the results of meta-analysis were stable.

## Discussion

The present study demonstrates that ESWT is significantly effective in reducing plaque size and relieving pain compared with the control group, whereas it is not effective in decreasing penile curvature and improving sexual function. This is consistent with the conclusion of the meta-analysis in 2016, but only three of the six included studies were high-quality RCTs, the low quality of which may had affected the accuracy of the final results. [19] In the same way, the meta-analysis in 2021 only believed that ESWT had a significant effect on reducing plaque size from the experimental point of view. Considering the heterogeneity of results caused by the differences in the natural history of disease of patients included in various experiments and inconsistent quantitative statistical criteria of clinical data (such as sexual function), the experiment abandoned the meta-analysis of some aspects, and still held a relatively conservative attitude towards ESWT as a treatment for PD. [20].

Previous studies have different opinions on the therapeutic effect of ESWT on PD. The prospective study of Husain et al. concluded that ESWT can improve penile curvature and reduce erectile pain; [18] a small single- arm trial by Kiyota et al. proposed ESWT as a surgical alternative in improving or even reversing the formation of fibrous plaques as well as reducing pain, but failed to find an improvement in penile curvature; [25] Manikandan et al. gave relatively optimistic conclusions that ESWT was effective in improving penile curvature and relieving pain during erection, but there was no significant evidence for improvement in erectile function. [26] Other studies hold a more conservative view. The prospective study conducted by Hauck et al. gave the opinion that ESWT only has a certain role in relieving pain, and cannot provide evidence as a standardized treatment scheme for PD. [11] In the study by Strebel et al., pain could be relieved to some extent in the majority of the population in which ESWT was applied, but other experimental results did not reach the expected degree of improvement, so they did not consider ESWT as a routine treatment, especially in patients who wanted to correct penile deformity and improve long-term sexual function. [13] Mortensen et al. studied the combined treatment of vacuum pump and ESWT, and finally recognized that ESWT is a non-invasive treatment with less side effects, but has no significant effect on the improvement of major symptoms. [27].

The present study, although somewhat optimistic in its results compared with previous published studies, still has certain limitations due to the low number of high-quality clinical studies that can be included at present. As there are differences in the expression methods adopted by the individual findings in terms of decreasing penile curvature (such as ventral or dorsal [22, 24]), we consider the results of the meta-analysis using continuous quantitative data to be heterogeneous and therefore do not adopt the method of the previous studies. [24] We simply defined the reduction of penile curvature in various directions as improvement, which resolved the controversy among multiple authors. We included the research results of Hatzichristodoulou [23] and Sokolakis [24] and analyzed using dichotomous outcome variables. As for the measurement results of the reduction of plaque size, only Palmieri’s study [21] provided continuous outcome data. Although the measurement methods adopted by different studies are different (such as ruler, ultrasound and palpation), it is a pity that the accurate area data of plaque size reduction cannot be obtained and the dichotomous data is used for analysis The 4 RCTs included in this study all used the standard VAS score scale to assess the degree of pain. We collected and supplemented relevant data according to the formula officially provided by Cochrane, and analyzed continuous outcome variables, which minimized the heterogeneity caused by missing data. However, the symptoms of PD have different manifestations at different times. As the disease progresses, the patient’s tolerance to pain and the way to assess pain will change, and may even be accompanied by spontaneous relief of pain, [10, 28] the patients included in this study are not completely consistent in the duration of symptoms, thus affecting the experimental results to a certain extent. For the assessment of “sexual function”, Palmieri’s [21] and Chitale’s [22] studies uniformly used IIEF-5 score scale for sexual function evaluation, while Hatzichristodoulou’s [23] study only used self-made questionnaires for subjective intention evaluation because the use of IIEF-5 has not been popularized, similar to sokolakis’ study. [24] Considering the widespread use of IIEF-5, it is inappropriate to define the degree of improvement in sexual function as a dichotomous outcome variable of subjective will. Therefore, we included two studies with IIEF-5 scores for the analysis of continuous outcome variables. In addition to considering the measurement method of the research results and the differences in the baseline data of the patients included, the lack of some research results and the differences in the follow-up time (4 weeks to 3 years) have become potential factors affecting the results of this study. In summary, the use of dichotomous outcome variables for analysis and the rational application of statistical formulas make up for the lack of continuous outcome data to a certain extent, but the lack of data accuracy is also one of the main sources of heterogeneity of results.

Analysis of complications is also a part worth discussing, which is an important perspective to prove the safety of ESWT. The 4 RCTs all paid attention to the occurrence of complications, which is worthy of reference for subsequent research. “Bruises” is a clearly reported complication after treatment. In our study defined the occurrence of “bruises” as postoperative complications with the exclusion of minor abrasions of the skin and small bleeding points. There are differences in the intensity of shock waves applied in different studies, and in course settings, leading to differences in the negative effects of shock waves on patients. It is also important to note when conducting intervention designs that, although increasing the intensity and frequency of shock waves within a certain range is able to increase efficacy, too high doses of shock waves also cause irreversible damage to penile structures, increase the risk of complications, and impair physiological function of PD patients, which has also been demonstrated in animal trials. [29] Fortunately, based on the analysis of the included studies, the complication rate is not significantly increased in the ESWT group compared with the control group, indicating that the application of low-dose shock waves can play a therapeutic role without causing serious complications.

Compared with previous studies, this study has included more literatures and all are high-quality RCTs. However, there are still differences among different studies in baseline data of patients, interventions as well as the way outcomes were evaluated, which partly affect the reliability of the results. In the follow-up study, the inclusion and exclusion criteria should be strictly set, and a relatively consistent experimental intervention plan (such as shock wave energy, frequency, etc.) should be developed, a unified evaluation method of outcome indicators (such as penile curvature - degree, plaque size - mm^2^, pain degree - VAS score, sexual function - IIEF-5 score), and similar follow-up time of outcomes should be developed. Similar suggestions were also given in the previous meta-analysis. [20] However, it is worth noting that the results of this study and previous similar clinical trials show that ESWT, as a physical therapy, can not fundamentally correct the penis deformity caused by tunica albuginea lesions, but can only alleviate the additional symptoms of the lesions, such as pain and inflammatory reaction. Therefore, we need more high-quality studies for exploring the application strategies of ESWT, and higher levels of evidence can be obtained through clinical experiments with uniform intervention conditions, which can guide us to give more effective recommendations for the clinical application of ESWT. For example, combination therapy with drugs such as antioxidants, rather than pursuing the efficacy of ESWT alone. [30, 31].

## Conclusions

ESWT has a certain effect on relieving pain during erection or sexual intercourse and softening or reducing plaques in the short term, but its effect on completely reversing the curvature of the penis and improving sexual function in the long term is still very limited. As a safe and effective treatment, it still deserves our attention. We need more high-quality clinical studies for more precise evaluation.

### Electronic supplementary material

Below is the link to the electronic supplementary material.

Supplementary Material 1

## Data Availability

All data generated or analysed during this study are included in this published article.
